# Lymphocyte–to–high–density lipoprotein ratio is negatively associated with diabetic macular edema in type 2 diabetic patients

**DOI:** 10.3389/fendo.2026.1828501

**Published:** 2026-05-01

**Authors:** Ying Yang, Yan Zhu, Dongmei Feng, Xiaoqun Wang

**Affiliations:** Department of Ophthalmology, The Affiliated Jiangning Hospital of Nanjing Medical University, Nanjing, China

**Keywords:** diabetic macular edema, diabetic retinopathy, immune-inflammation, lymphocyte-to-high-density lipoprotein ratio, type 2 diabetes

## Abstract

**Background:**

Systemic immune-inflammatory imbalance plays a potential role in the development of diabetic macular edema (DME). The lymphocyte-to-high-density lipoprotein ratio (LHR) can reflect the systemic immune-inflammatory status. This study aimed to explore the relationship between LHR and DME.

**Methods:**

This retrospective study enrolled 423 patients with diabetic retinopathy admitted to the same hospital between January 2022 and December 2024. Patients were categorized into DME (n = 163) and non-DME groups (n = 260) based on optical coherence tomography. Clinical and laboratory information was gathered, and LHR was calculated. Logistic regression models were conducted to explore the association between LHR and DME. Quartile grouping, quartile-based trend test, and subgroup analyses were used to verify the pattern of association. Restricted cubic splines (RCS) were used to assess nonlinear relationships. Sensitivity analysis was also conducted.

**Results:**

The DME group exhibited a significantly lower LHR level than in the non-DME group [1.55 (1.19, 2.04) vs. 1.94 (1.38, 2.47), P < 0.001]. Further adjusted regression models revealed that reduced LHR independently correlated with DME (aOR = 0.714, 95% CI: 0.541–0.942, P = 0.017). Quartile analysis demonstrated that the DME incidence in the Q1 group (LHR ≤ 1.32) was 48.1%, markedly higher than 27.6% in the Q4 group (LHR ≥ 2.40) (P = 0.002). RCS analysis verified a negative linear correlation between LHR and the odds of DME (P overall = 0.039, P nonlinear = 0.178). Sensitivity analysis supported the association (aOR = 0.698, 95% CI: 0.511–0.952, P = 0.023).

**Conclusion:**

Low LHR is independently associated with the presence of DME.

## 1 Introduction

Diabetic macular edema (DME) is a leading cause of vision loss among diabetic retinopathy (DR) patients (1). A range of treatments exist for DME including anti-vascular endothelial growth factor (anti-VEGF) and sustained-release glucocorticoids. However, 31.6%–65.6% of patients still suffer from persistent DME after standard treatment (2). With the worldwide increasing prevalence of diabetes, the management of DME poses a great challenge to public health.

In recent years, chronic low-grade inflammation has been widely verified to function critically in diabetic microvascular complications (3). Systemic inflammation gives rise to a pro-inflammatory and pro-thrombotic microenvironment via activated leukocytes and dyslipidemia, worsening intraocular inflammation, damaging the blood-retinal barrier, which may cause DME (4). Given the potential impact of systemic inflammation on DME, it is crucial to identify factors that accurately reflect the systemic immune-inflammatory status and are associated with DME.

Lymphocyte-to-high-density lipoprotein ratio (LHR) is a new immune-inflammatory composite index that has been reported to be associated with various diseases (5–8). Existing studies have confirmed the correlation between LHR and diabetic peripheral vascular diseases (9). But the role of LHR in diabetic microvascular complications is unclear. This study was designed to examine the correlation between LHR and DME in type 2 diabetes mellitus (T2DM).

## 2 Materials and methods

### 2.1 Study design and participants

**Figure 1:**
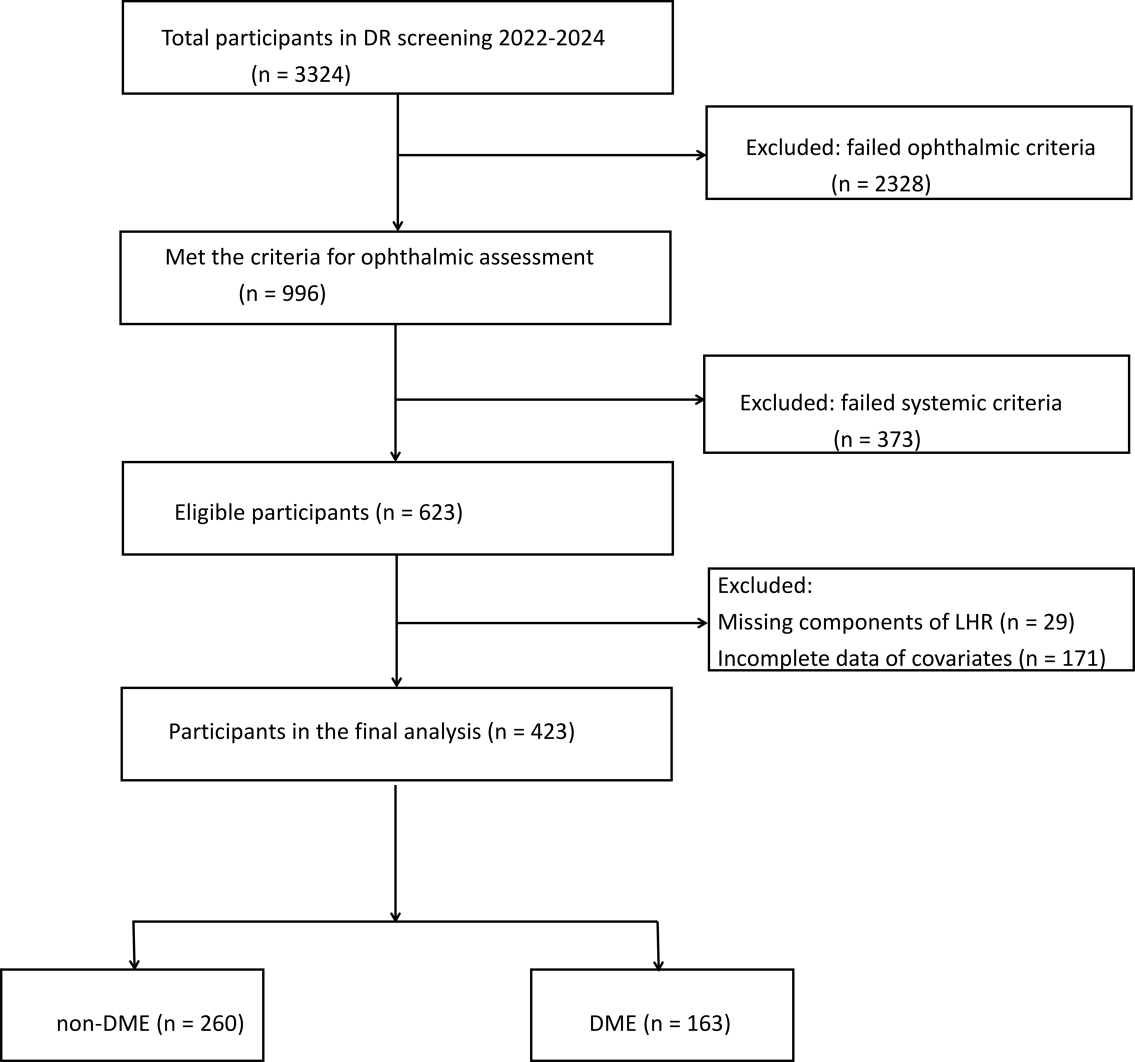
Selection flowchart of participants.

Medical records of diabetic patients undergoing fundus examination at our institution between January 2022 and December 2024 were retrospectively reviewed (**Figure 1**). Eligibility criteria: (1) Patients fulfilling the diagnostic criteria for T2DM (10); (2) Meeting the diagnostic criteria for DR and DME (11). Exclusion criteria: (1) Complicated with local or systemic infectious diseases; (2) Complicated with acute diabetic complications such as diabetic ketoacidosis; (3) Complicated with severe cardiac, hepatic or renal insufficiency, malignant tumors, hematologic diseases or immune system diseases; (4) Trauma or surgery within the prior 3 months; (5) Suspected subclinical infection, defined as a serum C-reactive protein (CRP) level >10 mg/L measured on the second morning after admission; (6) Receiving hemodialysis; (7) taking glucocorticoids or other immunomodulatory agents; (8) Complicated with other ocular diseases (e.g., glaucoma, uveitis, retinal vein occlusion, macular degeneration), or prior retinal laser therapy, retinal surgery, or intravitreal injection; (9) Incomplete data.

All patients underwent DR screening within 3 days of admission, including best-corrected visual acuity (BCVA), slit-lamp examination, intraocular pressure measurement (IOP), dilated funduscopy, fundus photography, and optical coherence tomography (OCT).

DME was diagnosed based on spectral-domain OCT (Heidelberg, Germany) if any of the following criteria was met: (1) intraretinal fluid, (2) subretinal fluid, (3) central retinal thickness (CRT) ≥ 300 μm. OCT images were acquired using a 512×128 scan mode with a 6×6 mm scanning area. CRT was manually measured using the built-in software of the OCT.

All image assessments were independently performed by two qualified ophthalmologists. In case of any discrepancies between the two examiners, a third senior ophthalmologist was consulted to resolve the differences and make the final judgment. All examiners were blinded to the patients’ clinical grouping and laboratory index during the entire image analysis process.

Only one eye per patient was included, with the more severely affected eye selected for analysis. This study finally included 423 patients. They were classified into two groups, non-DME group (n = 260) and DME group (n = 163). Missing values for each variate were summarized in the overall population and stratified by DME status (**Supplementary Table S1**). Baseline characteristics were also compared between included and excluded participants to assess potential selection bias (**Supplementary Table S2**).

### 2.2 Data gathering

Date were extracted from the hospital information system. Biological samples were collected on the morning after admission. Blood samples were obtained from the antecubital vein, following an overnight fast. Two-hour postprandial blood glucose was measured using venous blood obtained 2 hours after a standard meal.

All laboratory tests were performed according to standard clinical laboratory procedures. The normal reference ranges were as follows: hemoglobin A1c (HbA1c): 3.8–6.5%, Fasting blood glucose (FBG): 3.9–6.1 mmol/L, High-density lipoprotein (HDL): 1.16–1.42 mmol/L, Lymphocyte count: 1.1–3.2 ×10^9^/L, Neutrophil count: 1.8–6.3 ×10^9^/L, Monocyte count: 0.1–0.6 ×10^9^/L, Platelet count: 125–350 ×10^9^/L, C-reactive protein (CRP): 0.1–10 mg/L, Urinary albumin-to-creatinine ratio (UACR): 0–30 mg/g.

The following indices were calculated manually:

- LHR = lymphocyte count/HDL
- monocyte-to-high-density lipoprotein ratio (MHR) = monocyte count/HDL
- neutrophil-to-high-density lipoprotein ratio (NHR) = neutrophil count/HDL
- platelet-to-high-density lipoprotein ratio (PHR) = platelet count/HDL

Concomitant medications were recorded, including lipid−lowering agents (statins), antihypertensive agents (ACE inhibitors, ARBs, and others), antidiabetic agents (oral hypoglycemic agents, insulin, GLP−1 receptor agonists, and SGLT−2 inhibitors), and antiplatelet agents (aspirin and clopidogrel).

### 2.3 Statistical analysis

The Shapiro-Wilk test was employed to assess data normality. Data with normal distribution were presented as mean ± standard deviation (mean ± SD), and the independent samples t-test was utilized for between-group comparisons. For non-normally distributed data, they were reported as median with interquartile range (M [P25, P75]), and intergroup comparisons were carried out via the Mann-Whitney U test. Categorical variables were described as counts and percentages (n [%]), and the Chi-square test was adopted to compare differences between groups. Statistical analyses were performed using SPSS software (version 27.0; IBM, Armonk, NY, USA) and R software version 4.5.1with the RMS package. A P-value < 0.05 was regarded as statistically significant.

#### 2.3.1 Primary analysis

Multivariate binary logistic regression analyses were employed to explore the relationship between LHR and DME. Multiple models were constructed by sequentially adjusting for different variables. Model 1: unadjusted model; Model 2: adjusted for sociodemographic factors encompassing gender and age; Model 3: further adjusted for diabetes-related parameters to include duration of diabetes and hemoglobin A1c; Model 4: the fully adjusted model, which adjusted for Model 3 plus body mass index (BMI), diastolic blood pressure (DBP), total cholesterol, hypertension, lipid-lowering agents, DR stage, urinary albumin-to-creatinine ratio (UACR) and C-reactive protein (CRP).

#### 2.3.2 Comparison of LHR quartile groups

Four subgroups (Q1–Q4) were defined according to the LHR quartiles from the lowest to the highest. Differences in the incidence of DME across the four subgroups were examined using the Chi-square test.

#### 2.3.3 Quartile-based trend test

LHR quartile groups were included in model 4, with the highest quartile (Q4) set as the reference category. Adjusted odds ratios (aORs) and 95% confidence intervals (CIs) for Q1, Q2, and Q3 were calculated. A P for trend was estimated to assess the graded association between LHR levels and DME.

#### 2.3.4 Nonlinear dose−response analysis

Restricted cubic spline (RCS) analyses were performed to investigate the nonlinear relationship of LHR with DME in the entire study population and subgroups stratified by DR severity.

#### 2.3.5 Subgroup analysis

Subgroup analyses were conducted stratified by age (< 60 vs. ≥ 60 years), duration of diabetes (< 10 vs. ≥ 10 years), HbA1c (< 7.00% vs. ≥ 7.00%), BMI (< 24.00 vs. ≥ 24.00 kg/m²) and DR stage (non-PDR vs. PDR). The adjusted effect estimates (ORs and 95% CIs) for the relationship between LHR and DME were calculated in each clinical subgroup. To evaluate the modifying effect of stratified factors on this association, interaction terms (LHR × stratified factor) were entered into the regression model, and the P-values for interaction were calculated.

#### 2.3.6 Sensitivity analysis

Extreme outliers of continuous variables (LHR, HbA1c, DBP, BMI, and UACR) were excluded using the mean ± 3 standard deviations criterion. Multivariate logistic regression analysis was repeated with the remaining data, and the results were compared with the main findings to verify the stablility of the results.

## 3 Results

### 3.1 Comparison of baseline characteristics

The DME group exhibited significantly longer diabetic duration, higher HDL and lower BMI, diastolic blood pressure (DBP), lymphocyte number, LHR and PHR (all P < 0.05). The prevalence of DME was significantly higher in the PDR group than in the non-PDR group (χ² = 47.481, P < 0.001). The other variables did not differ significantly between groups (**Table 1**).

**Table 1:** Baseline characteristics of the two study groups.

variables	Non-DME (n = 260)	DME (n = 163)	Z/*χ²*	P
Age (years)	56.00 (48.25, 64.75)	58.00 (52.00, 66.00)	-1.567	0.117
Gender			1.213	0.271
Male [n(%)]	167 (64.23)	96 (58.90)		
Female [n(%)]	93 (35.77)	67 (41.10)		
Duration (years)	10.00 (7.00, 15.00)	10.00 (7.00, 17.00)	-2.234	0.025
BMI (kg/m²)	25.21 (23.15, 27.51)	24.80 (22.38, 26.70)	-2.042	0.041
SBP (mmHg)	139.00 (129.00, 151.00)	139.00 (127.00, 149.00)	-0.490	0.624
DBP (mmHg)	84.00 (77.00, 91.00)	80.00 (74.00, 86.00)	-3.416	< 0.001
HbA1c (%)	9.37 (8.08, 10.58)	9.37 (8.09, 10.95)	-0.038	0.970
FBG (mmol/L)	7.04 (5.74, 8.48)	7.05 (5.94, 8.76)	-0.962	0.336
2h-PBG(mmol/L)	16.41 (14.02, 18.31)	16.66 (14.65, 18.7)	-1.022	0.307
TC (mmol/L)	4.47 (3.77, 5.15)	4.27 (3.60, 5.25)	-0.776	0.438
TG (mmol/L)	1.38 (1.03, 2.20)	1.24 (0.90, 2.08)	-1.842	0.066
HDL (mmol/L)	0.89 (0.78, 1.10)	0.96 (0.81, 1.18)	-2.033	0.042
LDL (mmol/L)	2.72 (2.07, 3.42)	2.54 (1.93, 3.33)	-0.706	0.480
Scr (umol/L)	59.85 (47.43, 69.90)	62.20 (49.30, 75.50)	-1.504	0.133
CRP (mg/L)	0.66 (0.50, 1.87)	0.81 (0.50, 2.05)	-0.519	0.603
UACR (mg/g)	20.00 (5.00, 40.00)	20.00 (10.00, 60.00)	-0.519	0.222
Comorbidities				
Hypertension (n, %)	131 (50.38)	72 (44.17)	1.549	0.213
CHD (n, %)	22 (8.46)	9 (5.52)	1.275	0.259
CKD (n, %)	34 (13.08)	27 (16.56)	0.987	0.320
Concomitant medications				
LLA (n, %)	48 (18.53)	27 (16.56)	0.265	0.607
AHT (n, %)	111 (42.69)	58 (35.58)	2.111	0.146
ADA (n, %)	227 (87.31)	148 (90.80)	1.213	0.271
APT (n, %)	33 (12.69)	22 (13.50)	0.057	0.811
DR stage			47.481	< 0.001
Non-PDR (n, %)	244 (68.54)	112 (31.46)		
PDR (n, %)	16 (23.88)	51 (76.12)		
Neutrophil (10^9^/L)	3.80 (2.98, 4.70)	3.60 (3.00, 4.60)	-0.590	0.555
Lymphocyte (10^9^/L)	1.74 (1.40, 2.20)	1.61 (1.26, 2.00)	-2.839	0.005
Monocyte (10^9^/L)	0.40 (0.30, 0.50)	0.38 (0.30, 0.50)	-1.071	0.284
Platelet (10^9^/L)	194.50 (161.00, 227.00)	186.00 (156.00, 226.00)	-1.034	0.301
LHR	1.94 (1.38, 2.47)	1.55 (1.19, 2.04)	-3.817	< 0.001
MHR	0.40 (0.29, 0.60)	0.38 (0.25, 0.53)	-1.776	0.076
NHR	4.05 (3.04, 5.45)	3.88 (2.75, 5.29)	-1.488	0.137
PHR	208.78 (157.69, 263.40)	190.10 (153.25, 238.04)	-2.419	0.016

DME, diabetic macular edema; BMI, body mass index; SBP, systolic blood pressure; DBP, diastolic blood pressure; HbA1c, hemoglobin A1c; FBG, fasting blood glucose; 2h-PBG, 2-hour postprandial blood glucose; TC, total cholesterol; TG, triglyceride; HDL, high-density lipoprotein; LDL, low-density lipoprotein; Scr, serum creatinine; CRP, C-reactive protein; UACR, urinary albumin-to-creatinine ratio; CHD, coronary heart disease; CKD, chronic kidney disease; LLA: lipid-lowering agents; AHT, antihypertensive agents; ADA, antidiabetic agents; APT, antiplatelet agents; DR, diabetic retinopathy; PDR, proliferative diabetic retinopathy; LHR, lymphocyte-to-high-density lipoprotein ratio; MHR, monocyte-to-high-density lipoprotein ratio; NHR, neutrophil-to-high-density lipoprotein ratio; PHR, platelet-to-high-density lipoprotein ratio.

### 3.2 Primary analysis

The ORs were consistently less than 1 with P < 0.05 across Model 1 to Model 4 (**Table 2**).

**Table 2:** Association between LHR and DME in adjusted logistic regression models.

Models	B	SE	Wald	OR	95% CI		P
					Lower limit	Upper limit	
Model 1	-0.437	0.128	11.717	0.646	0.503	0.830	< 0.001
Model 2	-0.409	0.130	9.972	0.664	0.515	0.856	0.002
Model 3	-0.412	0.130	10.116	0.662	0.514	0.854	0.001
Model 4	-0.337	0.142	5.676	0.714	0.541	0.942	0.017

Model 1, unadjusted; Model 2, adjusted for gender and age; Model 3, adjusted for Model 2 plus duration of diabetes, hemoglobin A1c; Model 4, adjusted for Model 3 plus body mass index, diastolic blood pressure, total cholesterol, hypertension, lipid-lowering agents, DR stage, urinary albumin-to-creatinine ratio and C-reactive protein.

LHR, lymphocyte-to-high-density lipoprotein ratio; DME, diabetic macular edema; PHR, platelet-to-high-density lipoprotein ratio.

### 3.3 Comparison of LHR quartile groups

**Figure 2:**
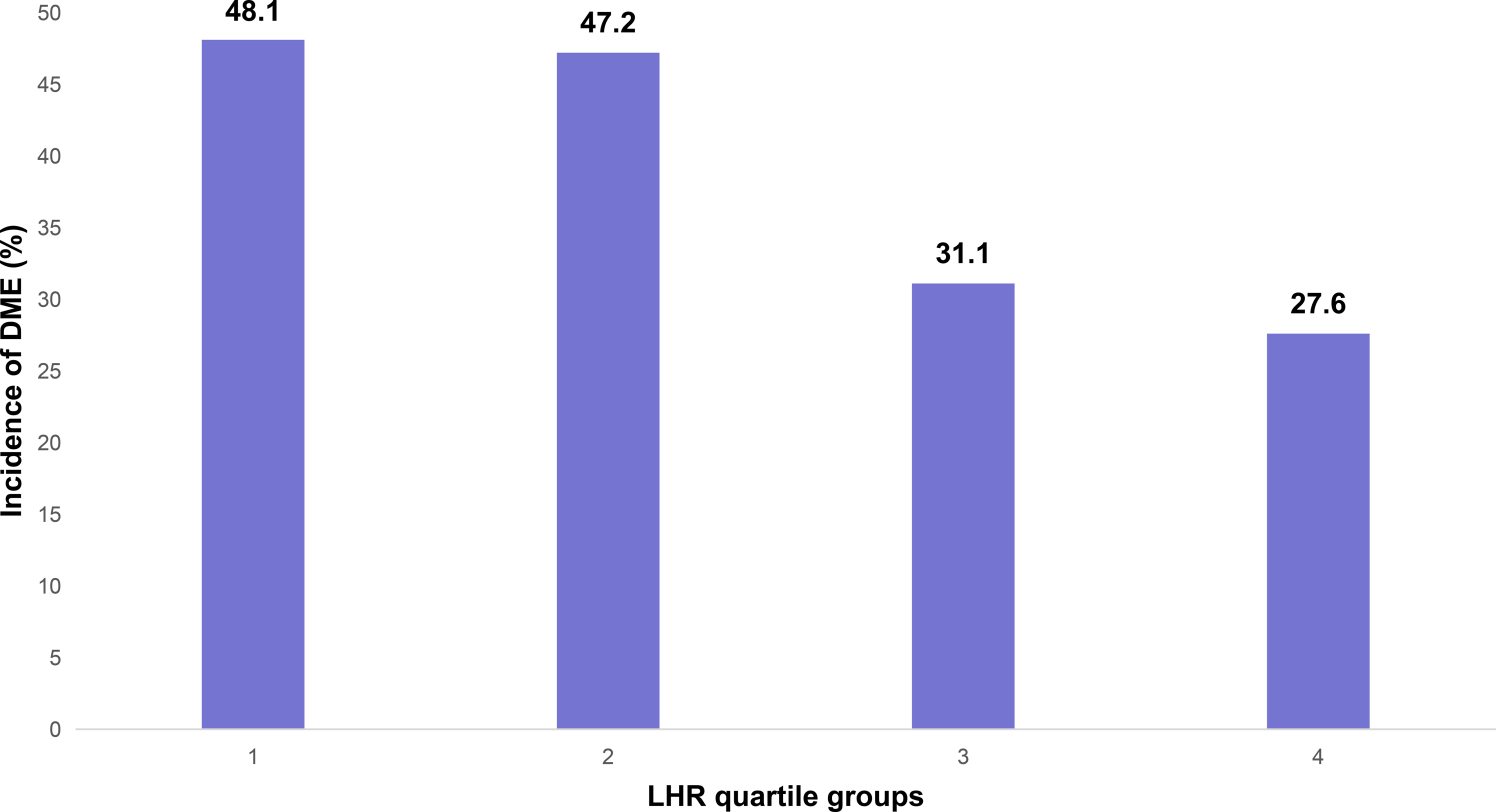
Incidence of DME across LHR quartile groups. LHR, lymphocyte-to-high density lipoprotein ratio; DME, diabetic macular edema; Q, quartile.

The incidence of DME differed significantly across the four LHR groups (Q1: ≤ 1.32; Q2: 1.33–1.75; Q3: 1.76–2.39; Q4: ≥ 2.40; χ² = 15.178, P = 0.002), with the highest rate observed in the Q1 group (**Figure 2**).

### 3.4 Quartile-based trend test

In Model 4, with LHR-Q4 as the reference, the adjusted ORs (95%CI) of DME were 1.880 (0.977–3.681), 1.829 (0.963–3.471), and 0.988 (0.511–1.910) for Q1, Q2 and Q3, respectively. LHR level was inversely associated with DME in overall population (OR = 0.777, 95% CI: 0.631–0.957, P for trend = 0.018). No consistent association was observed in the DR stage subgroups (**Table 3**).

**Table 3:** Quartile-based trend test for the association between LHR quartiles with DME risk.

LHR quartiles	Overall population aOR (95%CI)	Non-PDR subgroup aOR (95%CI)	PDR subgroup aOR (95%CI)
Q4 (≥ 2.40)			
Q3 (1.76–2.39)	0.988 (0.511, 1.910)	1.030 (0.511, 2.075)	0.279 (0.017, 4.660)
Q2 (1.33–1.75)	1.829 (0.963, 3.471)	1.629 (0.825, 3.217)	0.880 (0.052, 15.073)
Q1 (≤ 1.32)	1.880 (0.977, 3.681)	1.774 (0.881, 3.570)	1.977 (0.148, 26.360)
P for trend	0.018	0.056	0.310

LHR, lymphocyte-to-high-density lipoprotein ratio; DME, diabetic macular edema; aOR, adjusted odds ratio; CI, confidence interval; PDR, proliferative diabetic retinopathy; Q, quartile.

### 3.5 Nonlinear dose−response analysis

**Figure 3:**
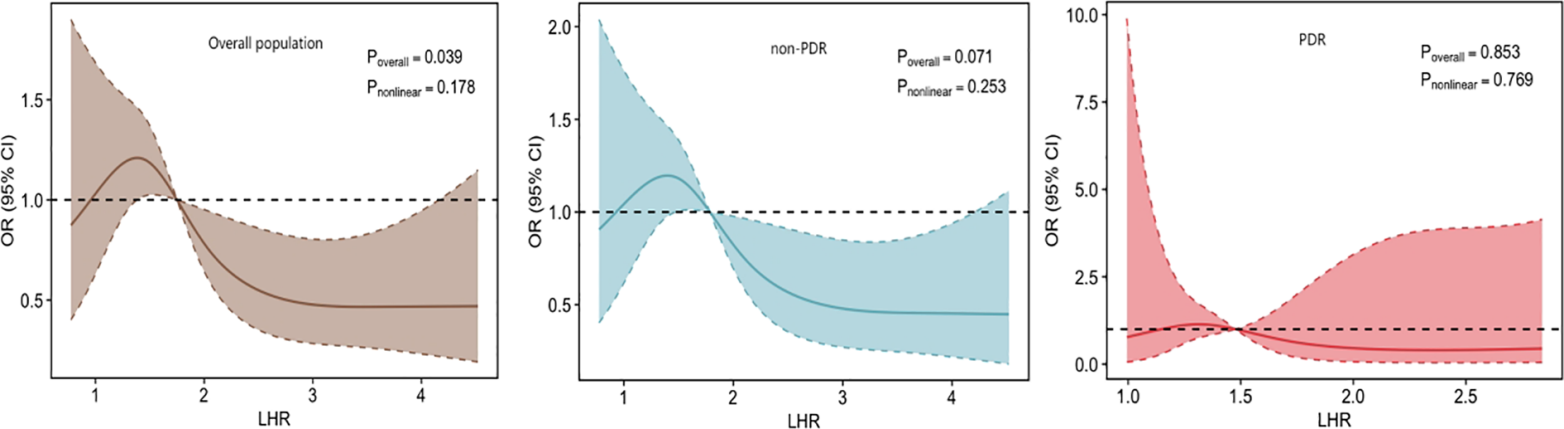
Nonlinear dose-response relationship between LHR and DME risk. Restricted cubic spline regression was used to adjust for gender, age, diabetes duration, hemoglobin A1c, body mass index, diastolic blood pressure, total cholesterol, hypertension, lipid-lowering medication use, severity of diabetic retinopathy, urinary albumin-to-creatinine ratio, and C-reactive protein to evaluate the dose-response relationship.

In the overall population, a significant negative linear relationship was found between LHR and DME (P overall = 0.039, P nonlinear = 0.178). The odds ratio (OR) of DME decreased steadily with increasing LHR levels. In the non-PDR subgroup, a marginally significant negative linear trend was consistent with the overall population (P overall = 0.071, P nonlinear = 0.253). No significant association between LHR and DME was found in the PDR subgroup (P overall = 0.853, P nonlinear = 0.769) (**Figure 3**).

### 3.6 Subgroup analysis

Significant inverse associations between LHR and DME risk were detected in the subgroups of age < 60 years, diabetes duration ≥ 10 years, HbA1c ≥ 7.00%, BMI < 24.00 kg/m², and non-PDR subgroup (all P < 0.05), whereas no significant association was found in the opposite subgroups (all P > 0.05). None of the stratified factors showed a significant interactive effect (all P for interaction > 0.05) (**Table 4**).

**Table 4:** Subgroup analysis of the association between LHR and DME risk.

Character	n	OR (95% CI)	P	P for interaction
Age (years)				0.415
< 60	249	0.714 (0.541, 0.942)	0.017	
≥ 60	174	0.964 (0.660, 1.407)	0.848	
Duration (years)				0.552
< 10	162	0.699 (0.421, 1.158)	0.164	
≥ 10	261	0.698 (0.490, 0.994)	0.046	
HbA1c (%)				0.103
< 7.00	43	1.272 (0.145, 11.146)	0.828	
≥ 7.00	380	0.711 (0.531, 0.951)	0.022	
BMI (kg/m²)				0.258
< 24.00	154	0.401 (0.206, 0.778)	0.007	
≥ 24.00	269	0.852 (0.618, 1.174)	0.328	
DR stage				0.560
Non-PDR	356	0.740 (0.553, 0.990)	0.042	
PDR	67	0.631 (0.227, 1.751)	0.377	

LHR, lymphocyte-to-high-density lipoprotein ratio; DME, diabetic macular edema; HbA1c, hemoglobin A1c; BMI, body mass index; DR, diabetic retinopathy; PDR, proliferative diabetic retinopathy; OR, odds ratio; CI, confidence interval.

### 3.7 Sensitivity analysis

After excluding 29 participants with extreme outliers, a total of 394 patients remained. The results (aOR = 0.698, 95% CI: 0.511–0.952, P = 0.023) were consistent with those of the main analyses (aOR = 0.714, 95% CI: 0.541–0.942, P = 0.017).

## 4 Discussion

LHR reflects two pathophysiological processes: lymphocyte-mediated immune activation and HDL-mediated lipid metabolism disorder (12). It was initially used to study metabolic syndrome and insulin resistance, and was later applied to the assessment of psychiatric, cardiac and other related diseases (5, 13–16). However, its correlation with DME remains unclear. To our knowledge, we are the first to report that low LHR is independently associated with DME in patients with T2DM, and a negative linear relationship exists between LHR levels and the presence of DME. This association showed stable and consistent across multiple adjusted models, stratified analyses and sensitivity analyses.

In previous studies, VEGF and chronic inflammation have been the primary research focuses of DME. However, a considerable proportion of patients still show inadequate responses to both anti-VEGF and anti-inflammatory therapies (2, 17). The pathogenesis of DME may be far more complex than the known mechanisms. In our study results, lymphocyte counts were significantly lower in DME patients relative to non-DME patients (P = 0.005), which is in accordance with that reported by Do et al. (18). Lymphocytes are critical effector cells in the adaptive immune response, and abnormalities in their quantity and function can directly lead to immune imbalance (19). Retinal homeostasis also relies on lymphocyte-mediated adaptive immunity (20). Persistent hyperglycemia in patients with T2DM triggers intracellular oxidative stress and induces lymphocyte apoptosis through advanced glycation end products (AGEs) accumulation, resulting in an immunosuppressive state in the body (21, 22). The pathological link of lymphopenia-immunosuppression-retinal homeostatic imbalance may serve as a new complementary mechanism for the occurrence of DME.

Traditional views hold that HDL exerts reverse cholesterol transport and anti-inflammatory effects, playing a protective role (23). Low HDL levels are linked to the development of DR (24, 25). However, the protective effects of HDL are not static; its functions are dynamically altered by the body’s metabolic and inflammatory status. Accumulating evidence indicates that HDL exhibits anti-inflammatory properties under healthy conditions, whereas it exerts pro-inflammatory effects during chronic inflammatory states like T2DM (26, 27). In the DR patients enrolled in this study, the median HDL levels in both groups (0.96 mmol/L in the DME group vs. 0.89 mmol/L in the non-DME group) were slightly below the local laboratory reference range (1.16–1.42 mmol/L). However, DME patients showed slightly higher HDL levels than non-DME patients (P = 0.042). This finding appears to contradict the traditional protective role of HDL. We speculate that the elevated HDL levels in DME patients may represent a compensatory response under chronic hyperglycemia stress. Although the body attempts to increase HDL to counteract vascular injury, such HDL often becomes dysfunctional under hyperglycemia and chronic inflammation, losing its protective anti-inflammatory properties (28). Previous studies have demonstrated that elevated HDL levels cannot effectively reduce the risk of cardiovascular events, and dysfunctional HDL may even exert pro-atherogenic effects (29). These mechanisms may partly explain the HDL paradox observed in the present study, suggesting that dysfunctional HDL may play an important role in DME. This hypothesis warrants further investigation.

As a systemic composite index of immune inflammation, the LHR integrates lymphocytes and HDL, and thus can provide a more comprehensive reflection of potential immune-inflammatory imbalance (7). The reduced LHR in DME patients may result from immune suppression caused by lymphopenia and HDL dysfunction. These two pathological changes ultimately promote the onset and progression of DME by disrupting retinal homeostasis and exacerbating the intraocular inflammatory microenvironment. Existing evidence has confirmed that low LHR is closely linked to unfavorable outcomes in various inflammatory diseases. For instance, septic patients with low LHR have an increased 90-day mortality rate; low LHR is associated with impaired lung function; hepatitis B patients with low LHR have a poor prognosis; and low LHR is linked to increased all-cause mortality risk in asthmatic populations (6, 23, 30, 31). These studies collectively indicate that a decreased LHR level can reflect systemic immune-inflammatory imbalance and dysfunction, providing important theoretical support for the association between low LHR and DME observed in the present study.

In this study, we also compared the baseline levels of other HDL-related inflammatory indices. We found that PHR levels were lower in DME patients than in non-DME patients, whereas NHR and MHR levels were similar between the two groups. Currently, studies on the associations of these three indices with DR or DME remain limited, with inconsistent results reported in the literature. Yalinbas et al. reported elevated MHR levels in DME patients, while Tang et al. reported similar MHR levels between DME and non-DME patients (32, 33). As far as we are aware, this is the first study to demonstrate an independent inverse association between LHR and DME, offering new sights into the pathogenesis of DME.

In the baseline data analysis of the present study, the two groups also differed in terms of BMI and DBP. The association between BMI and DME remains controversial in the existing literature. Li et al. reported that median BMI differed significantly between DME and non-DME group (23.72 vs. 24.38 kg/m^2^) (34). This finding is consistent with our results. By contrast, Haliyur et al. failed to identify a significant association between BMI and DME (35). DBP was lower in DME group in our study, which is inconsistent with the findings of previous research (36). These discrepancies may be due to distinct characteristics of the study populations. After adjusting for all the above factors in the final multivariate model, LHR remained stably and independently associated with DME.

In the present study, exploratory subgroup analyses were employed to further investigate the association between LHR and DME. Although significant inverse associations were observed in certain subgroups, no significant interaction was detected for any stratified factor (all P for interaction > 0.05). These inconsistent results may be attributed to the limited sample size and potential residual confounding factors that were not fully controlled for. The results should be interpreted with caution.

This study has several inherent limitations. This single-center retrospective design may be associated with inherent selection bias. The lack of follow-up data precluded prognostic assessment. We did not measure key inflammatory cytokines such as IL-6, TNF-α, or VEGF, which limits direct evidence supporting the underlying inflammatory mechanism. This study was conducted in an inpatient setting, which may lead to non−negligible selection bias.

## 5 Conclusion

In this study, we comprehensively described and analyzed the association between LHR and DME. We demonstrated for the first time that a low LHR is correlated with higher odds of DME. This finding may improve our understanding of the pathogenesis involved in DME. Further research is required to verify our findings and develop the potential clinical value.

## Data Availability

The raw data supporting the conclusions of this article will be made available by the authors, without undue reservation.
