# Supplementary material for: Lymphocyte–to–high–density lipoprotein ratio is negatively associated with diabetic macular edema in type 2 diabetic patients
Source: Front Endocrinol (Lausanne). 2026 May 1;17:1828501. doi: 10.3389/fendo.2026.1828501 (PMC13175880; doi:10.3389/fendo.2026.1828501)
Supplement: Supplementary file 1 [file Table1.doc]

Table S1: Missing data of covariates among 623 participants

| Variable | Over (n = 623) | Non-DME (n = 391) | DME (n = 232) |
| --- | --- | --- | --- |
|  | Missing (n, %) | Missing (n, %) | Missing (n, %) |
| Age (years) | 0 (0) | 0 (0) | 0 (0) |
| Gender | 0 (0) | 0 (0) | 0 (0) |
| Duration (years) | 22 (3.53) | 14 (3.58) | 8 (3.45) |
| BMI (kg/m²) | 34 (5.46) | 21 (5.37) | 13 (5.60) |
| SBP (mmHg) | 11 (1.77) | 7 (1.79) | 4 (1.72) |
| DBP (mmHg) | 11 (1.77) | 7 (1.79) | 4 (1.72) |
| HbA1c (%) | 31 (4.98) | 19 (4.86) | 12 (5.17) |
| FBG (mmol/L) | 0 (0) | 0 (0) | 0 (0) |
| 2h-PBG(mmol/L) | 21 (3.37) | 13 (3.32) | 8 (3.45) |
| TC (mmol/L) | 29 (4.65) | 18 (4.60) | 11 (4.74) |
| TG (mmol/L) | 29 (4.65) | 18 (4.60) | 11 (4.74) |
| HDL (mmol/L) | 29 (4.65) | 18 (4.60) | 11 (4.74) |
| LDL (mmol/L) | 29 (4.65) | 18 (4.60) | 11 (4.74) |
| Scr (umol/L) | 25 (4.01) | 16 (4.09) | 9 (3.88) |
| CRP (mg/L) | 17 (2.73) | 11 (2.81) | 6 (2.59) |
| UACR (mg/g) | 32 (5.14) | 20 (5.12) | 12 (5.17) |
| Comorbidities |  |  |  |
| Hypertension (n, %) | 0 (0) | 0 (0) | 0 (0) |
| CHD (n, %) | 0 (0) | 0 (0) | 0 (0) |
| CKD (n, %) | 0 (0) | 0 (0) | 0 (0) |
| Concomitant medications |  |  |  |
| LLA (n, %) | 0 (0) | 0 (0) | 0 (0) |
| AHT (n, %) | 0 (0) | 0 (0) | 0 (0) |
| ADA (n, %) | 0 (0) | 0 (0) | 0 (0) |
| APT (n, %) | 0 (0) | 0 (0) | 0 (0) |
| DR stage |  |  |  |
| Non-PDR (n, %) | 0 (0) | 0 (0) | 0 (0) |
| PDR (n, %) | 0 (0) | 0 (0) | 0 (0) |
| Neutrophil (109/L) | 17 (2.73) | 11 (2.81) | 6 (2.59) |
| Lymphocyte (109/L) | 17 (2.73) | 11 (2.81) | 6 (2.59) |
| Monocyte (109/L) | 17 (2.73) | 11 (2.81) | 6 (2.59) |
| Platelet (109/L) | 17 (2.73) | 11 (2.81) | 6 (2.59) |
