# Supplementary material for: Lymphocyte–to–high–density lipoprotein ratio is negatively associated with diabetic macular edema in type 2 diabetic patients
Source: Front Endocrinol (Lausanne). 2026 May 1;17:1828501. doi: 10.3389/fendo.2026.1828501 (PMC13175880; doi:10.3389/fendo.2026.1828501)
Supplement: Supplementary file 2 [file Table2.doc]

Table S2: Baseline characteristics between included and excluded participants

| Variable | Included (n = 423) | Excluded (n = 200) | Z/χ² | P |
| --- | --- | --- | --- | --- |
| Age (years) | 57.00 (50.00, 65.00) | 55.00 (50.00, 63.00) | -1.243 | 0.214 |
| Gender |  |  | 0.102 | 0.750 |
| Male (n, %) | 263 (62.17) | 127 (63.50) |  |  |
| Female (n, %) | 160 (37.83) | 73 (36.50) |  |  |
| FBG (mmol/L) | 7.04 (5.80, 8.50) | 7.20 (5.80, 9.01) | -1.016 | 0.310 |
| Comorbidities |  |  |  |  |
| Hypertension (n, %) | 203 (47.99) | 84 (42.00) | 1.961 | 0.161 |
| CHD (n, %) | 31 (7.33) | 16 (8.00) | 0.088 | 0.767 |
| CKD (n, %) | 61 (14.42) | 35 (17.50) | 0.988 | 0.320 |
| Concomitant medications |  |  |  |  |
| LLA (n, %) | 75 (17.77) | 23 (11.50) | 4.022 | 0.045 |
| AHT (n, %) | 169 (39.95) | 69 (34.50) | 1.710 | 0.191 |
| ADA (n, %) | 375 (88.65) | 167 (83.50) | 3.187 | 0.074 |
| APT (n, %) | 55 (13.00) | 25 (12.50) | 0.031 | 0.861 |
| DR stage |  |  | 9.924 | 0.002 |
| Non-PDR (n, %) | 356 (84.16) | 147 (73.50) |  |  |
| PDR (n, %) | 67 (15.84) | 53 (26.50) |  |  |
| DME presence (n, %) | 163 (38.53) | 68 (34.00) | 1.197 | 0.274 |
